# Operative Dentistry

**Published:** 1883-01

**Authors:** 


### Operative Dentistry.

By J. Taft.

The fourth edition of this work is just issued by P. Blakiston, Son & Co., Philadelphia.

In the preparation of this as in the former editions, the aim and effort have been to eliminate that which was obsolete, or has become supplanted, and to introduce the new and the improved; indeed, to bring the work, so far as possible, up to the present time. To accomplish either of these absolutely is, of course, utterly impossible. Since that which is best with some is far from being the best, or even good, in the hands of others. The time devoted to the work was shorter than the author could have wished. In the preparation of this edition the aim has been to bring the work more nearly to meet the requirements of the student than in the other issues. How nearly these objects have been attained the author must leave others to judge.

To know that the mechanical execution and dress of the work are all that could be expected or desired it is sufficient to say that P. Blakiston, Son & Co., Philadelphia, are the publishers.

